# Inflammation decreases keratin level in ulcerative colitis; inadequate restoration associates with increased risk of colitis-associated cancer

**DOI:** 10.1136/bmjgast-2014-000024

**Published:** 2015-05-18

**Authors:** Bernard M Corfe, Debabrata Majumdar, Arash Assadsangabi, Alexandra M R Marsh, Simon S Cross, Joanne B Connolly, Caroline A Evans, Alan J Lobo

**Affiliations:** 1 Molecular Gastroenterology Research Group, Academic Unit of Surgical Oncology, Department of Oncology, University of Sheffield, The Medical School, Sheffield, UK; 2 Insigneo Institute for *in silico* Medicine, University of Sheffield, Sheffield, UK; 3 Gastroenterology Unit, Royal Hallamshire Hospital, Sheffield, UK; 4 Academic Unit of Pathology, Department of Neuroscience, Faculty of Medicine, Dentistry & Health, University of Sheffield, Sheffield, UK; 5 Waters Corporation, Wilmslow, UK; 6 Biological and Systems Engineering Group, Department of Chemical and Biological Engineering, ChELSI Institute, University of Sheffield, Sheffield, UK; 7 Gastroenterology Unit, Royal Hallamshire Hospital, Sheffield, UK

**Keywords:** CYTOKERATINS, DYSPLASIA, GUT INFLAMMATION, ULCERATIVE COLITIS

## Abstract

**Background:**

Keratins are intermediate filament (IF) proteins, which form part of the epithelial cytoskeleton and which have been implicated pathology of inflammatory bowel diseases (IBD).

**Methods:**

In this study biopsies were obtained from IBD patients grouped by disease duration and subtype into eight categories based on cancer risk and inflammatory status: quiescent recent onset (<5 years) UC (ROUC); UC with primary sclerosing cholangitis; quiescent long-standing pancolitis (20–40 years) (LSPC); active colitis and non-inflamed proximal colonic mucosa; pancolitis with dysplasia-both dysplastic lesions (DT) and distal rectal mucosa (DR); control group without pathology. Alterations in IF protein composition across the groups were determined by quantitative proteomics. Key protein changes were validated by western immunoblotting and immunohistochemical analysis.

**Result:**

Acute inflammation resulted in reduced K8, K18, K19 and VIM (all p<0.05) compared to controls and non inflamed mucosa; reduced levels of if– associated proteins were also seen in DT and DR. Increased levels of keratins in LSPC was noted relative to controls or ROUC (K8, K18, K19 and VIM, p<0.05). Multiple K8 forms were noted on immunoblotting, with K8 phosphorylation reduced in progressive disease along with an increase in VIM:K8 ratio. K8 levels and phosphorylation are reduced in acute inflammation but appear restored or elevated in subjects with clinical and endoscopic remission (LSPC) but not apparent in subjects with elevated risk of cancer.

**Conclusions:**

These data suggest that keratin regulation in remission may influence subsequent cancer risk.

**Summary box**

**What is already known about this subject?**

▸  Ulcerative colitis (UC) is associated with an increased risk of colitis-associated cancers; factors such as severity of colonic inflammation (endoscopic and histological), duration of disease and concomitant primary sclerosing cholangitis are associated with increased risk.
▸  Keratins are a type of intermediate filament (IF) proteins, which, as part of cellular cytoskeleton have important regulatory functions on the colonic mucosa. Keratin 8 (K8) null mice develop colitis and K8 is shown to modulate tumour necrosis factor action. Missense mutations in the keratin 8 gene have been noted in a subset of patients with inflammatory bowel diseases.
▸  Vimentin, a type III IF protein is often considered as a canonical marker of epithelial, mesenchymal transformation. Increased levels have been noted in aggressive colorectal cancers.

**What are the new findings?**

- Proteomics demonstrate that acute inflammation results in reduction in keratins 8, 18, 19 (K8, K18, K19) and vimentin (VIM) levels.
- Phosphorylated K8 (K8pS23) levels relative to total K8, is increased in controls and long-standing pancolitis, while a reduced ratio is seen in dysplasia.
- There is also a presence of low molecular weight K8 forms and reduced K8 phosphorylation in inflamed mucosa compared to proximal uninflamed colon. Further proteomic analysis has shown potential differences between these proteoforms, suggesting potential value as biomarkers.

**How might it impact on clinical practice in the foreseeable future?**

- Keratin (K) levels and phosphorylation are reduced in acute inflammation, but restore or increase following clinical and endoscopic remission. This process may be impaired in patients who have an elevated risk of cancer suggesting that keratin regulation in remission may be a pivotal factor influencing subsequent risk of development of colitis-associated cancer. Monitoring of relative ratio of vimentin to K8, phosphorylated K8, or appearance of novel proteoforms might be useful as markers of aggressive disease phenotype in ulcerative colitis.

## Introduction

Intermediate filaments (IF) are an important component of the cellular cytoskeleton, with keratins constituting the major IF proteins in epithelial cells and accounting for approximately 5% of total cellular protein.^1^ ^2^ In the intestinal epithelium, principally expressed keratins are keratins 8, 18 and 19 (K8, K18, K19).^2^ Vimentin (VIM), a type III IF protein is primarily found in cells of mesenchymal origin^3^ and replaces keratins early in epithelial–mesenchymal transition. IF proteins share a similar structure.^2^ The structure, solubility and functions of IFs are regulated, in part, by a range of post-translational modifications (PTMs), including phosphorylation, glycosylation, acetylation and cleavage.^2^ ^4^ Specific sites of phosphorylation, predominantly in the head and tail domains, have been noted in serine (S) residues of K8 at S23, S73 and S431. Phosphorylation of these sites is under control of protein kinases including p38,^5^ mitogen-activated protein kinases (MAPK)^6^ and c-jun kinase.^7^ The curated PTM deposition database, phosphosite (http://www.phosphosite.org), indicates additional PTM types including acetylation and glycosylation. Sumoylation, which exhibits reciprocity with acetylation at some sites, has also been demonstrated to modify K8 function in liver.^8^ IFs are dynamic and undergo reorganisation in response to a variety of stimuli including, mitosis, apoptosis and other cellular stresses often with redistribution between an insoluble (filamentous) and a soluble pool.^9^

Keratins are associated with the pathogenesis of various colorectal diseases, including cancer and inflammatory bowel diseases (IBD). They have been shown to be critical in maintaining the epithelial integrity and to protect against mechanical and non-mechanical stresses,^10^ and regulate effects of signalling pathways. Keratins also function in cell-death signalling pathways, in particular apoptosis mediated by Fas and tumour necrosis factor (TNF) α.^11^ ^12^ Stromal VIM expression in colorectal cancers correlates with malignant potential of the tumours.^13^

The pathogenesis of ulcerative colitis (UC) is still not fully understood, and factors such as altered immune response to luminal microbiota^14^ and alterations in mucosal barrier function^15^ may be contributory. A potential role of impaired epithelial barrier function due to alterations in keratin levels has been hypothesised.^16^ Notably, the keratin gene superfamily is located within the IBD2 locus on chromosome 12.^16^ Heterozygous missense mutations in the K8 gene have been reported in patients with IBD with in vitro experiments demonstrating inefficient filament assembly by the mutant K8 in comparison with wild type.^17^ K8-deficient mice (mK8^−/−^FVB/N) develop colonic inflammation and mucosal hyperplasia;^18^ a mucosal chronic T-helper type 2 inflammatory response has been noted in K8^−/−^ mice that develop colitis.^19^ The latter change reverses with antibiotic therapy suggesting a contributory role of luminal bacterial microbiota.^20^

UC elevates risk of developing colitis-associated cancer (CAC). Duration of colitis is an important factor in increasing colon cancer risk, with cumulative colorectal cancer (CRC) incidence noted at 2%, 8% and 18%, at 10, 20 and 30 years, respectively.^21^ Extent of colonic involvement,^22^ concomitant primary sclerosing cholangitis (PSC)^23^ as well as early age of onset of UC are also risk factors for CAC.^22^ Severity of inflammation increases the risk of dysplasia and CRC,^24^ and both microscopic as well as inflammation evident endoscopically are considered important.^25^ ^26^

Quantitative proteomic techniques enable not only identification of proteins in biological samples, but also their alteration in level between samples.^27^ This permits determination of changes in response to disease-related factors. Few studies have investigated mucosal proteomic changes in IBD, and only one has shown changes in cytoskeletal proteins, showing elevated levels of actin cytoskeletal and associated proteins in patients with a higher risk of UC progression.^28^ Given associations between K8 and colorectal cancer progression and its interactions with inflammatory pathways, we investigated the mucosal changes in IFs in patients with UC with differing cancer risk and inflammatory status.

## Materials and methods

### Patients

Patients (18–75 years old) with histologically proven UC and healthy controls (CON) were recruited prospectively from outpatient clinics and inpatient wards at the Royal Hallamshire Hospital, Sheffield, UK. The study was approved by South Yorkshire Research Ethics Committee (10/H1310/21). Written informed consent was obtained from all patients. Clinical activity in patients with UC was defined by the Baron UC disease activity index score.^29^

Patients were categorised into groups of differing CRC risk and inflammatory status.

1. Recent onset (<5 years duration) UC (ROUC).
2. Long-standing pancolitis (LSPC)—with disease duration between 20 and 40 years.

Patients in the LSPC and ROUC groups were in established deep remission with no evidence of endoscopic activity (Baron endoscopic scores 0 and 1, respectively) and no microscopic activity (histological activity scores 0 in both groups).

1. UC with concomitant primary sclerosing cholangitis (PSC);
2. Dysplasia in patients with pancolitis (dysplastic tissue, DT);
3. Rectal mucosa distant to areas of dysplasia (DR).

Biopsies were obtained in patients with active distal colitis from

1. The inflamed rectal mucosa (ACTively inflamed mucosa; ACT);
2. The proximal, endoscopically uninflamed mucosa from the same patients as 6 (INACT);
3. CONs were patients undergoing lower gastrointestinal (GI) endoscopic examination which was endoscopically and histologically normal, with no prior history of IBD, nor from hospital records did they develop IBD in the 2 years after biopsies were obtained.

Biopsies were obtained at the rectum from all the patients with UC in remission as well as normal controls.

### Lower GI endoscopy and colonic biopsy

All patients underwent flexible sigmoidoscopy or colonoscopy following bowel preparation using either Kleanprep (Norgine Limited, UK) or Picolax (Ferring Pharmaceuticals, UK). Endoscopic biopsies were collected from the colon using Radial Jaw 4, biopsy forceps (Boston Scientific Corporation, Massachusetts, USA).

Biopsies were snap-frozen in liquid nitrogen and stored at −80°C (for proteomic analyses) or formalin fixed for histological assessment. Exclusion criteria and patient data are in the online supplementary data.

### Preparation of IF-enriched fraction

We followed our technique for isolation and solubilisation of IFs for mass spectrometry (MS).^30^ The IF fraction from cultured MCF-7 cells used as controls in the western immunoblot and as internal standards for densitometry.^30^ Experimental details are provided in full (see online supplementary data, sections 3 and 5).

### Proteomic methods

*iTRAQ (isobaric tags for relative and absolute quantitation) protein profiling*: Briefly, pooled samples were tryptically digested, labelled with 8-plex iTRAQ reagents, separated by strong cation exchange offline before tandem MS. Peptides were identified with Phenyx and Uniprot, before relative quantification and statistical analysis.^31^ Details are in the online supplementary data, section 4A.

*Label-free MS*: Gel bands were excised and digested in-gel prior to LC/MS-MS analysis coupled with ion mobility (HDMS^E^). Data processing used UniProt and Progenesis QI software.^32^ Details are in the online supplementary data, section 4B.

### Sodium dodecyl sulfate polyacrylamide gel electrophoresis and western immunoblotting

Buffer exchange, western transfer and immunoblotting were as previously described.^4^ Primary antibodies were mouse monoclonal: anti-K8 (ab9023); K18 (ab668), K19 (ab7754); K8 phospho-specific rabbit antiphospho S73 antibody (ab32579) and phospho S431 (ab59434) (supplied by Abcam, Cambridge, UK). In-house antibodies raised against acetylated lysine10 residue of K8 (rabbit).^33^ Densitometric analysis is detailed in online supplementary data, section 7. Immunoblotting was performed for (n=56) patients.

### Histological assessments of inflammation and keratin expression

Biopsies were formalin fixed, and tissue sections (4 µm thick) were cut. Sections were H&E stained, and reviewed by a single histopathologist (SSC) blinded to the diagnosis. Dysplasia and histological severity of inflammation were defined according to standard criteria.^26^ ^34^ Immununohistochemistry of K8, K18 and K19 was essentially as described using antibodies to K8, K19 ab9023, ab7754, respectively (Abcam, Cambridge, UK) and K18 (in-house monoclonal antibody). Scoring of keratin for crypt intensity, crypt depth and surface intensity were as previously described.^35^ Immunohistochemistry (n=48, was performed where formalin-fixed paraffin embedded (FFPE) tissue was adequately well oriented for scoring).

### Statistical analysis

Data have been described as median and range/interquartile range, as indicated. Differences between groups were evaluated with the non-parametric Mann-Whitney U test. Analysis of iTRAQ data and relative quantification of fold changes in protein levels was performed as described previously^31^ where the criteria for alterations on protein level were p value <0.05, with Bonnferroni multiple test correction applied to reduce false positives.

## Results

### Patients and workflow

*Patients*: 62 adult patients with histologically proven UC healthy CONs were recruited prospectively from outpatient clinics or inpatient wards at Royal Hallamshire Hospital, Sheffield, UK. CONs were patients undergoing lower GI endoscopic examination who had normal endoscopic and histological appearance of the colonic mucosa. Patients were stratified into groups based on cancer risk. Inflammatory status measured by the Baron endoscopic appearance and the Histological Activity Score is detailed in the Methods section and in the online supplementary table S1.

### Acute inflammation results in reduced levels of keratins and VIM

**Figure 1:**
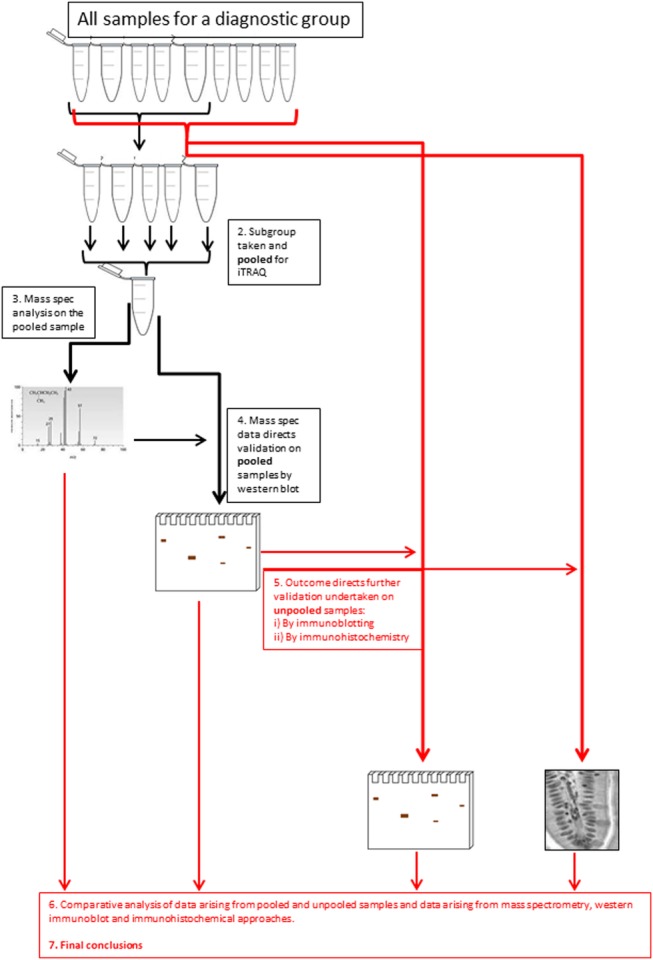
Graphical summary of the workflow. Protein profiles of samples from the eight-patient group were compared using iTRAQ (isobaric tags for relative and absolute quantitation)-based quantitative proteomics. Downstream validation was undertaken using both immunoblotting (n=56) and immunohistochemistry (n=48, as not all formalin-fixed paraffin embedded (FFPE) tissue was adequately well oriented for scoring).

**Figure 2:**
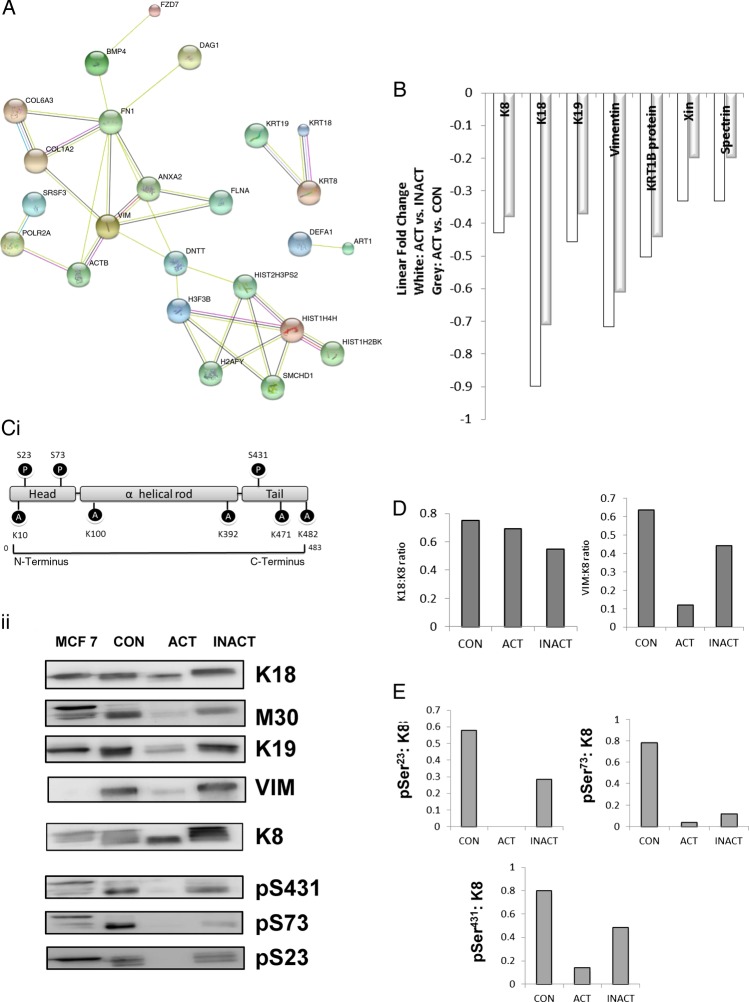
Effect on active inflammation on intermediate filament (IF) proteome. IF extracts were subject to proteomic analysis (see online supplementary data, sections 4 and 6). (A) Strings analysis of the global proteome showing key protein interacting networks (PINs). (B) Proteins identified included K8, K18, K19 and VIM (vimentin), as well as IF-associated proteins spectrin β chain brain 4, Xin actin-binding protein 1 and KRT1B as well as histone proteins and collagen. White bars show change in ACT relative to INACT, and grey bars showing ACT relative to control (CON). (Ci) Position of key antibodies used in this study and (Cii) immunoblots of ACT and INACT pooled samples entered into the proteomic analysis. Densitometry was undertaken and (D) ratios of Krt8 and either Krt18 or VIM; (E) alteration in relative phosphorylation at each site.

The insoluble proteome was extracted from flash-frozen biopsies and analysed using an iTRAQ workflow (figure 1 and online supplementary data, section 2). The demographic summary of samples used in the pooling is in online supplementary data, section 6. The list of proteins identified and relatively quantified in the analysis is detailed in the online supplementary data, section 6. In total, 53 proteins were identified and relatively quantified, of which 31 had information from two or more peptide sequences. A qualitative analysis of the whole dataset was undertaken using STRINGS, a database of known and predicted protein interactions to determine protein interacting networks (PINs; figure 2A) represented by the data set. Two principle PINs emerged in this analysis, with clusters around fibronectin (FN1) and the major keratin components K8, K18, K19 indicating linkage to extracellular matrix, consistent with IF function. The proteomic data for ACTively inflamed mucosa (ACT) and unINvolved mucosa in subjects with ACTive inflammation (INACT) were compared to identify alterations in protein levels between groups. Keratins were significantly decreased, with fold change in levels of K8 (0.4-fold), K18 (0.7-fold), K19 (0.4-fold) and VIM (0.6-fold) (all p<0.05) in inflamed mucosa in comparison with CONs as well as to the uninvolved proximal colon (figure 2B). The uninflamed mucosa did not show any significant difference in protein levels by MS from the mucosa of controls. Immunoblotting was undertaken to validate the proteomic data and, using a bank of antibodies to K8 and key post-translational modifications (figure 2Ci), to explore further the nature of changes in K8 associated with inflammation. The positions of the pSer^23^, pSer^73^ and pSer^431^ are shown relative to AcLys^10^ (subject to western blot, shown in the context of previously identified^4^ lysine acetylation sites; figure 2Ci). The western blot data is shown in figure 1Cii. Total K8 levels were reduced in ACT, both in comparison with CON and INACT; in ACT, K8 was predominantly concentrated in the lower molecular weight forms (37 kDa). A reduced ratio of Vim:K8 was also noted in ACT and not paralleled in changed ratios between K8 and K18 (figure 2D). Phosphorylation of K8 is reduced in tumour progression.^36^ The relative phosphorylation at sites pSer^431^, pSer^23^ and pSer^73^ in INACT, ACT and control tissues was therefore evaluated by western immunoblot and relative densitometry to an internal standard of MCF-7 IF extract (figure 2Cii, E). The data suggest that there is much less relative phosphorylation at all three sites (ie, when differential levels of K8 are controlled for) in the samples from inflamed colon, and that this is more pronounced in the inflamed versus the non-inflamed region, suggesting that a progressive dephosphorylation of K8 is associated with inflammatory status.

As the pooled samples used in iTRAQ and immunoblot may mask heterogeneity. We undertook two tiers of orthogonal validation of unpooled samples (see online supplementary data, section 8). Antibody-based methods of dot blotting and immunohistochemistry (IHC) were employed.

**Figure 3:**
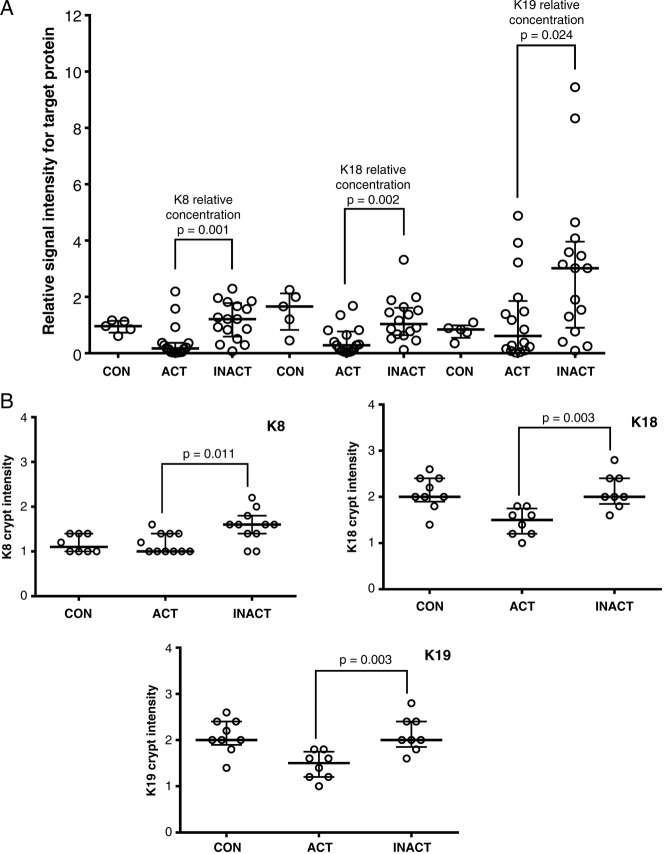
Analysis of individual variation in unpooled samples in response to inflammation. (A) Relative signal intensity differences for keratins 8, 18 and19 in colonic biopsies from patients with active colitis (ACT), and reciprocal proximal inactive colonic mucosa (INACT) from the same individuals in comparison with MCF-7 as an external control. Relative signal intensity differences for keratins 8, 18 and 19 from rectal colonic biopsy for five normal controls (CON) relative to MCF-7 are also shown. Solid horizontal lines represent the median with vertical bars as IQR in each group. Differences are tested using a Mann-Whitney U test. Raw data are shown in the supplementary online information. (B) Corresponding formalin-fixed paraffin embedded (FFPE) sections are stained and scored for keratins identified in this study. Colonic epithelial average crypt intensity for K8, K18 and K19 using immunohistochemical staining comparing patients with active colitis (ACT) with reciprocal proximal inactive colonic mucosa (INACT) from the same individuals; FFPE rectal tissues from normal individuals are IHC stained for K18 as CON; solid horizontal lines represent the median with vertical bars as IQR.

Figure 3A summarises by-patient changes in keratins assessed by dot-blotting for all three major keratins. Medians and distribution patterns are shown and agree with the pooled data (for raw data, see online supplementary data, section 8). Immunohistochemistry of keratin levels in crypts adds spatial information on the distribution within tissue, with the caveat that it is less sensitive, and the need to select scorable crypts potentially biases results. Analysis of distribution of keratins expression (figure 3B and see online supplementary figures S8.1, S8.2) showed significant changes in the intensity of staining in the crypt, as opposed to total intensity or depth of staining, agreed with the proteomic and immunoblot data.

### Long-standing quiescent disease associates with a distinct keratin expression and phosphorylation profile compared with either recent onset or higher cancer risk groups

**Figure 4:**
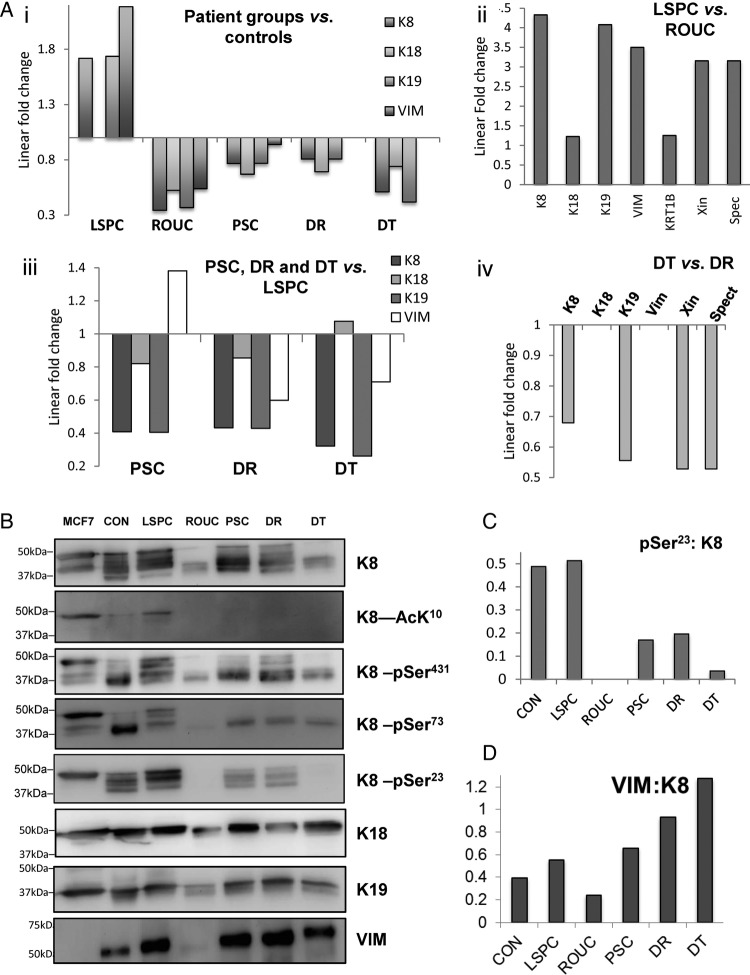
Proteomic analysis revealling intermediate filament (IF) proteins are differentially altered in ulcerative colitis (UC) subgroups. (A) iTRAQ (isobaric tags for relative and absolute quantitation) data of the changes in IF in patients with long-standing pancolitis (LSPC), recent onset UC (ROUC) and primary sclerosing cholangitis (PSC), and those with dysplasia (DR and DR, respectively). By comparison with controls (Ai), LSPC showing a significant increase in levels of K8, K19 and VIM (vimentin); in ROUC, PSC, DR and DT the levels of K8, K18 and K19 are significantly decreased. The oppositional direction of change between LSPC and other groups when set against controls reinforces evidence that this is a biological not a technical event. A comparison of major proteins in quiescent and LSPC disease was undertaken (Aii): LSPC samples exhibited a marked increase in all IF proteins and IF-associated proteins (Spectrin, Xin) compared with ROUC. Comparison of samples from higher risk subjects (PSC, DR, DT) against LSPC revealed marked changes in K8, K19 and VIM, with the VIM directionality distinguishing PSC from DT/DT. Finally in (Aiv) DT samples showed reduced levels of K8, K18, Xin and Spectrin relative to DR. (B) Results of orthogonal validation undertaken by western immunoblot using specific antibodies to K8, K18, K19 and VIM. Multiple isoforms of K8 are noted in all the patient groups including healthy controls with lower levels and low molecular weight forms predominating in ROUC and DT. Immunoblotting using specific antibodies to three major phosphorylation sites of K8 (pS23, pS73 and pS431) and one acetylation site of K8 (AcLys10) was undertaken, along with immunoblotting for K8 in the same samples. A control MCF-7 sample was included in all immunoblots to allow normalisation between experiments. Bands were quantified by densitometry, normalised within-blot to the MCF-7 control, and then across-blot to the K8 sample. Relative phosphorylation levels and VIM:K8 thereby determined are shown in (C and D).

The expression levels of key IF proteins (K8, K18, K19 and VIM) in the iTRAQ data set were compared across the patient groups in this study (figure 4A). Pooled samples entered into the proteomic analysis were subject to orthogonal validation by western immunoblot (figure 4B). When the control group was the normalising reference point (figure 4Ai), the analysis revealed partitioning of profiles: LSPC exhibited generally higher levels of IF proteins relative to control, whereas ROUC, PSC, DR and DT samples all showed reduced levels of most IF proteins relative to control, a marked opposition of direction of change. The increases noted in LSPC were significant: K8 and K19 (1.7-fold each, p<0.05) and VIM levels (2.2-fold) (figure 4Ai, B). By contrast, in ROUC, despite having macroscopically and microscopically quiescent disease, exhibited significantly reduced keratin levels relative to controls (K8, K18, K19 and VIM 0.3, 0.5, 0.4 and 0.5-fold respectively, all p<0.05). Coexisting PSC results in a fourfold increase in the colon cancer risk in colitis patients;^23^ likewise, we assessed tissue from patients with dysplasia both at the DT and uninvolved rectal mucosa (DR). In PSC, there was a significant reduction in keratin and VIM levels (0.8, 0.7, 0.8 and 0.9-fold for K8, K18 and K19 and VIM, respectively; p<0.05, figure 4Ai) in comparison with CON (figure 4Ai, B). Analysis of DT and rectal mucosa (DR) also showed a similar pattern of changes in DR and DT relative to controls (figure 4Ai, B). These changes suggest a possible pan-colonic field change in IF proteome alterations in patients with dysplasia.

In order to investigate effects of disease duration on IF proteins, LSPC data were compared with ROUC data (ROUC as reference set, figure 4Aii). In comparison with ROUC, a significant increase in levels of IF proteins (4.3, 1.2, 4 and 3.5-fold for K8, K18, K19 and VIM, respectively; p<0.05), and IF-associated proteins Xin and Spectrin was noted in LSPC (figure 4Aii), suggesting an effect of duration of disease on levels of principal keratins. The LSPC group exhibited oppositionality of changes for most keratins by comparison with the control group through a re-plot of the data comparing ROUC, PSC, DR and DT with LSPC as reference group (figure 4Aiii, B). In PSC, there was a significant reduction in keratin and VIM levels (0.8, 0.7, 0.8 and 0.9-fold for K8, K18 and K19 and VIM, respectively; p<0.05, figure 4Ai; online supplementary table S2) in comparison with the CON group (figure 4Ai, 2B). Analysis of DT relative to LSPC revealed a significant reduction in levels of IFs (0.3, 0.2 and 0.7-fold for K8, K19 and VIM, p<0.05) (figure 4Aiii), as well as KRT1B, Xin and Spectrin (figure 4Aiii;), 0.6-fold for both, p<0.05). Rectal mucosa (DR) also showed reduced levels (figure 3Aiv) relative to LSPC. Finally, in order to establish whether there were changes associated with dysplasia which may discriminate DR and DT tissue, a direct comparison was undertaken (figure 4Aiv) which revealed reduction of K8, K19, Xin and Spectrin in dysplastic tissue relative to matched controls.

As keratin phosphorylation in inflamed tissue was significantly reduced (vide supra), we sought to establish whether any such changes associate with particular subgroups of UC. Pooled samples, as entered into the proteomic analysis, were immunoprobed for K8, for phosphorylation at pSer^23^, pSer^73^, pSer^431^ and lysine acetylation at AcLys^10^ (figure 4B). Multiple immunoreactive bands of K8 corresponding to as many as six discrete bands between 37 and 50 kDa on western blot were noted in control patients, LSPC, PSC and DR (figure 3B). By contrast with controls, the isoforms in LSPC were evident at higher molecular weights, whereas in PSC and DR they tended to predominate at lower molecular weights. Similar pattern of K8 alteration is noted in quiescent ROUC mucosa as well as in DT. In order to assess whether these K8 forms arose through differential reversible post-translational modifications, we undertook western blot to identify common PTMs in K8 in the form of phosphorylation at pSer^23^, pSer^73^, pSer^431^ and lysine acetylation at AcLys^10^. In healthy controls, intense phosphorylation at Ser73 and Ser 431 was noted predominantly at a single band around 37 kDa, while phosphoSer23 antibody cross-reacted with a more diverse set of molecular weights. The pattern (if not the intensity) of cross-reaction was broadly conserved in LSPC and DR. DT and ROUC samples exhibited lower levels of K8 cross-reaction, a less diverse array of molecular weights, and so had correspondingly fewer cross-reactions with anti-PTM antibodies. LSPC, by contrast, exhibited cross-reactions for PTMs across a wider range of molecular weights and was the only in vivo sample to exhibit cross-reaction for lysine^10^. Densitometry allowed the quantitative assessment of changes in relatively phosphorylation, corrected for K8 level (see figure 3C and online supplementary data, section 3). The analysis reveals that relative phosphorylation in all sites is consistent between LSPC and CON, but lower in other conditions, markedly so at pSer^23^. As VIM levels were reduced in acute inflammation and in ROUC, and were increased in LSPC, PSC, DR and DT, a densitometry analysis (see online supplementary data, section 7 for regions quantified) of VIM and K8 immunoblots was undertaken (figure 4D). We undertook a novel analysis of the ratio of VIM to K8 which showed reduced levels in ROUC, in comparison with CONs. There was a progressive increase in VIM/K8 ratios from LSPC to PSC, with markedly increased levels noted in DT and DR (other comparisons were undertaken; see online supplementary data).

**Figure 5:**
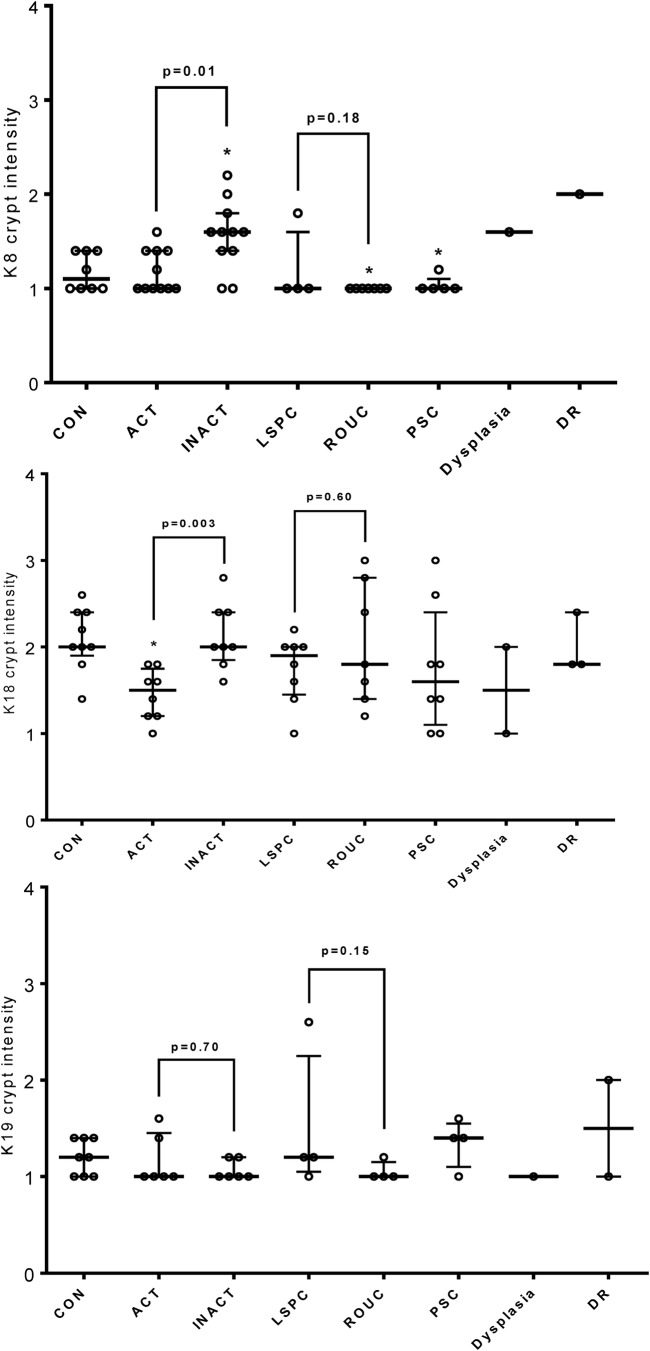
Analysis of individual variation in unpooled samples in response to inflammation. Corresponding formalin-fixed paraffin embedded (FFPE) sections were stained and scored for keratins identified in this study. Colonic epithelial average surface and crypt intensity for K8 (top panel), K18 (centre panel) and K19 (bottom panel) were scored comparing patients with: active colitis (ACT), reciprocal proximal inactive colonic mucosa (INACT) from the same individuals, long-standing pancolitis (LSPC), recent-onset ulcerative colitis (UC) (ROUC), UC with concomitant primary sclerosing cholangitis (PSC), UC with dysplasia, and rectal sample from UC with dysplasia (DR); FFPE rectal tissues from normal individuals were immunohistochemical staining for each keratin as control (CON). Each group was compared with the CON and the statistically significant difference marked with a (*) sign at the top. *p<0.05, Mann-Whitney U test.

Orthogonal validation of changes in levels of the major keratins in individual patients was undertaken as for ACT/INACT samples by IHC. This complementary methodology is considerably less sensitive than iTRAQ or western blot, but provides an assessment of total keratin as well as information changes in keratin level across the tissue architecture. Fold-changes observed relative to control samples were modest at the iTRAQ level (<2-fold for keratins in LSPC; <0.5-fold for keratins in other groups) and were not found to be significant (figure 5A), however, the general trends observed matched those identified by protein chemistry. It is likely that this also reflects that relatively fewer samples were scorable by IHC, and numbers in some pools were low.

### Mass spectrometric analysis of proteoforms of keratins identifies candidate biomarkers

**Figure 6:**
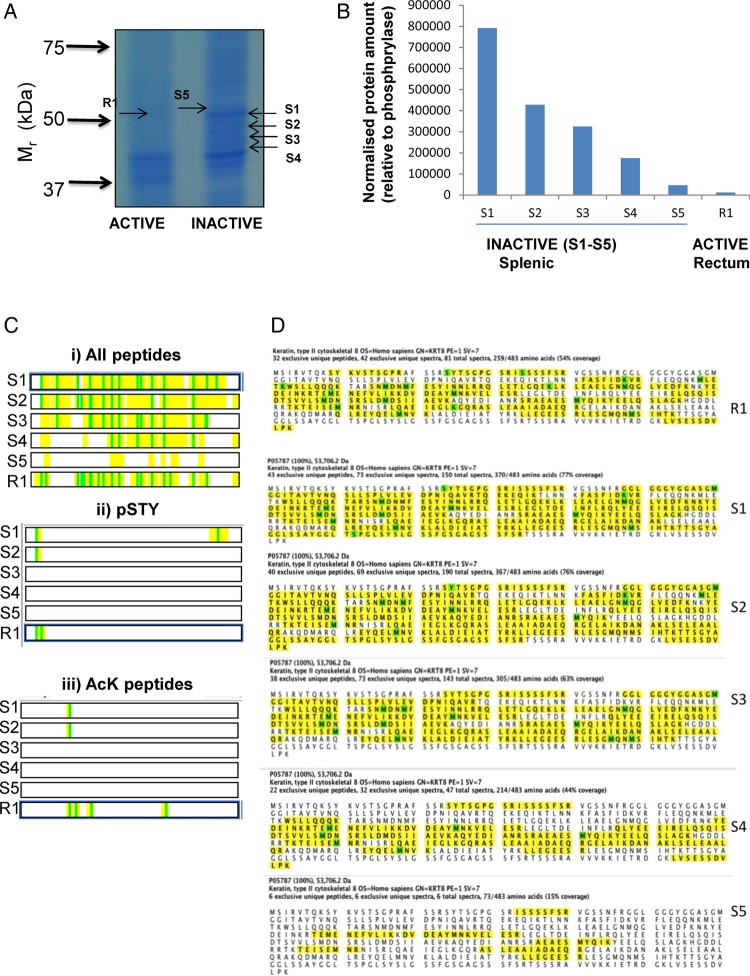
Analysis of keratin proteoforms using a label-free proteomic approach. (A) Proteins from intermediate filament (IF) fractions derived from biopsy materials from two patients with active ulcerative colitis (UC) and paired inactive biopsy material were resolved by sodium dodecyl sulfate polyacrylamide gel electrophoresis (SDS-PAGE; 10%). Bands in the region corresponding to K8 were excised from active (R1) and inactive (S1–S5) gel lanes. (B) The amount of K8 protein in each band was calculated. All samples were run in duplicate and the results are the mean values. (C) All peptides corresponding to K8, are shown in yellow for each sample with modified residues shown in green (5Bi), and (ii) phosphorylated and (iii) acetylated residues were identified and indicated in green. (D) The sequence coverage is shown for K8 in the gel bands analysed, peptides shown in yellow with modified residues oxidised methione, acetylated lysine or phosphoserine highlighted in green.

Immunoblot analysis using K8-specific antibodies revealed a series of immunoreactive bands: potentially representing different proteoforms (representing different splice variants and/or PTM status) of K8 (figure 2Cii, 4B). In order to investigate this further, paired IF fractions from distal, active, inflamed rectal mucosa, and from proximal, uninflamed mucosa in the same patient were studied. Patient samples were first resolved by sodium dodecyl sulfate polyacrylamide gel electrophoresis (SDS-PAGE; figure 5A). Individual bands (R1, S1-S5) were excised from the gel, proteolytically digested with trypsin, and analysed using label-free MS^37^ (see online supplementary data, section 4B). While all bands excised contained K8, this was not the predominant species for all, and relative recovery of K8 is shown in figure 6B. PTMs were identified: lysine acetylations at sites were detected at AcLys^107^, AcLys^122^, AcLys^158^, AcLys325 and phoshorylation of pSer^23^, pSer^34^ and pSer^431^ which represent the major phosphorylations sites of K8. The deconvoluted peptide maps of each species are shown in figure 6C and sequence coverage mapped in figure 6D. The data indicate a substantially reduced complexity of K8 proteoforms in active colitis.

## Discussion

Development of colon cancer in UC is multifactorial, and inflammation is now considered to play a significant role in its pathogenesis.^25^ The natural history of UC is variable: the frequency of disease flares is unpredictable and cancer develops in a small proportion of such patients. Ongoing active histological inflammation has been shown to predispose to development of CAC.^26^ In view of the evidence implicating inflammation in cancer and colitis, and the potential association of keratins in the colonic inflammation and the pathogenesis of IBD,^17^ ^18^ we investigated proteomic changes in IFs in well-characterised groups of patients with UC at varying levels of cancer risk. We have shown that acute inflammation is associated with a reduction in levels of keratins in the inflamed colonic mucosa, whereas the levels in the uninflamed proximal mucosa parallel those of healthy colonic mucosa in control patients. Keratins are dynamic and are involved in a range of inflammatory pathways, in particular protection from apoptosis mediated by TNF-α and Fas, with epithelial cells lacking K8/K18 being prone to apoptosis, and K8^−/−^ mice exhibiting hyperplasia, fragile mucosa and impaired ion transport.^11^ ^12^ ^38^ Our data show that keratin levels are at, or slightly higher than, normal level in patients with persistent quiescent disease, but that in active inflammation and in quiescent subjects with recent disease, there is a reduced level of keratin. Taken together, these data suggest a delay in the restoration of keratins in the mucosa following acute inflammation despite macroscopic and microscopic mucosal healing. Previous studies have also shown persistent cellular and molecular damage with activated kinase and transcription factor signalling pathways in the colon despite apparent microscopic healing.^39^

Phosphorylation of K8 affects its solubility and function.^2^ Reduced phosphorylation of K8 due to increased phosphatase of regenerating liver-3 expression is associated with colorectal cancer progression.^36^ Our data show reduced or a loss of phosphorylation in acute inflammation, with similar changes persisting in ROUC as well as DT. This suggests a protective effect of physiological phosphorylation of K8 at pSer^23^ would be lost in ROUC and DR/DT. Increased levels and phosphorylations of keratins in LSPC may be significant: LSPC is believed to be an important factor contributing to the pathogenesis of CAC, but levels of keratins in quiescent LSPC points towards a protective mechanism in this situation. The environmental and regulatory factors governing K8/K18 are not well characterised and merit analysis in future studies. There are limited data on the role of acetylation in regulation of keratin function, but we have previously shown association between Lys483 acetylation and depolymerisation. We noted that the K8 recovered from inflamed tissue appeared more extensively acetylated (by number of sites, not necessarily stoichiometry) which may suggest progression to entropy of IF and consequent plasticity of cells.

**Figure 7:**
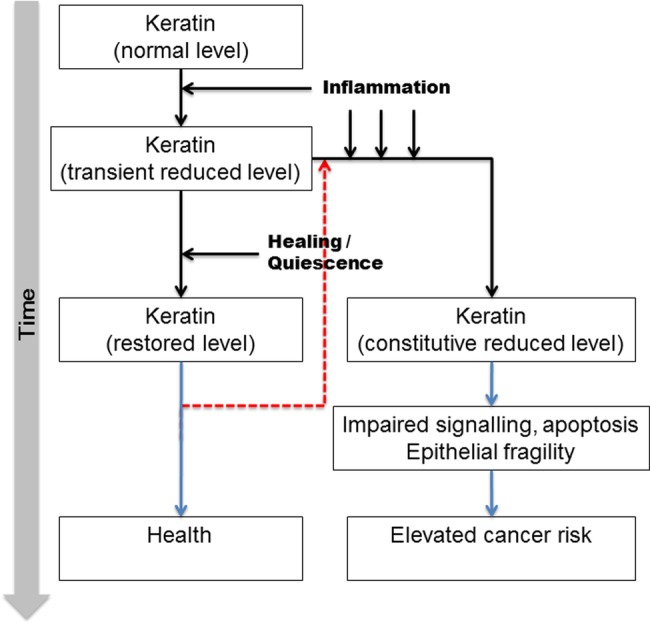
Proposed model of involvement of intermediate filament (IF) proteins in pathogenesis of dysplastic changes in ulcerative colitis (UC). A small number of patients despite an initial acute episode have long-standing quiescent disease process; these patients are at a lower risk of colorectal cancer (CRC) in spite of having a long-standing disease process. On the contrary, recurrent bouts of acute inflammation and inadequate recovery/restoration of keratins following the acute inflammation may contribute to pathogenesis of CRC. Some patients may exhibit inflammation even after restoration of keratin, and so re-enter the disease path (red dashed line).

Taken together with the results of acute inflammation on keratins, we can suggest that inadequate restoration of keratin expression following acute inflammation may contribute to the pathogenesis of CAC by affecting the repair process in the mucosa. This is supported by the changes seen in patients with PSC and dysplasia, but the number of patients in these groups is small. The findings, therefore, need confirmation in prospective studies in these subgroups. This study remains cross-sectional, and future prospective analyses will reveal whether repeated inflammation or impaired keratin expression is the more causal factor in CAC risk phenotypes. In this study, we show an acute reduction in VIM levels in the acutely inflamed mucosa as well as in ROUC. Increased expression of VIM, generally expressed in cells with a mesenchymal phenotype in LSPC may reflect morphological colonic tissue remodelling and architectural alterations, reflecting the chronic relapsing/remitting course of the disease as a consequence of accumulated damage during each active phase.^40^ ^41^ The VIM:K8 ratio progressively increases with development of a more aggressive phenotype, potentially due to epithelial denudation and crypt shortening associated with disease. These results suggest that relative overexpression of VIM in the colonic mucosa may herald the development of CAC. The data are integrated into a qualitative model for CAC progression in figure 7.

This is the first study investigating changes in insoluble IF levels in the mucosa in patients with UC with differing disease phenotype. Unlike previous studies which have focused on the soluble fraction of proteins, we have undertaken subcellular fractionation to investigate changes in the relatively insoluble fraction which, under physiological states, constitutes the bulk of the keratin pool. Our data suggest a model for the pathogenesis of CAC whereby acute inflammation reduces keratin levels and affects mucosal IF protein integrity which lags behind apparent clinical, microscopic and endoscopic recovery. Persistent failure of such recovery may be the cornerstone of pathogenesis of CAC. Additionally, the role of altered VIM:K8 ratio is significant and it might have a potential role as a mucosal marker of progressive disease. Nevertheless, prospective studies are needed, as are development of targeted strategies to modulate IF expression in the colonic mucosa.

## Supplementary Material

Supplementary Materials
