# Supplementary material for: Inflammation decreases keratin level in ulcerative colitis; inadequate restoration associates with increased risk of colitis-associated cancer
Source: BMJ Open Gastroenterol. 2015 May 18;2(1):e000024. doi: 10.1136/bmjgast-2014-000024 (PMC4599170; doi:10.1136/bmjgast-2014-000024)
Supplement: Supplementary Materials [file bmjgast-2014-000024.html]

Inflammation decreases keratin level in ulcerative colitis; inadequate restoration associates with increased risk of colitis-associated cancer: BMJ Open Gastroenterology: Vol 0, No 0

Online Supplement
