# Supplementary material for: Inflammation decreases keratin level in ulcerative colitis; inadequate restoration associates with increased risk of colitis-associated cancer
Source: BMJ Open Gastroenterol. 2015 May 18;2(1):e000024. doi: 10.1136/bmjgast-2014-000024 (PMC4599170; doi:10.1136/bmjgast-2014-000024)
Supplement: Supplementary Materials [file bmjgast-2014-000024supp.pdf]

Supplementary Online Information

***for*:**

Inflammation decreases keratin level in ulcerative colitis; inadequate post-inflammatory restoration associates with increased risk of colitis-associated-cancer

Bernard M Corfe[1], Debabrata Majumdar[1,2], Arash Assadsangabi[1,2], Alexandra MR Marsh[1], Simon S Cross[3], Joanne B. Connolly[4] Caroline A Evans[5], Alan J Lobo[2,6]

Author for Correspondence: Professor Alan J. Lobo, Gastroenterology Unit, P Floor, Royal Hallamshire Hospital, Beech Hill Road, Sheffield S10 2JF

Email: alan.lobo@sth.nhs.uk

Fax: 01142712396

Section 1 Exclusion Criteria

1. Patients unable to give signed informed consent
2. Non-correctable coagulopathy (prothrombin time > 12 seconds / platelet count < 90 x 109 / L)
3. Pregnant patient
4. Inadequate bowel preparation
5. Severe colitis or toxic dilatation of the colon or clinical condition where colectomy is highly likely
6. Patients with a diagnosis of indeterminate colitis, Crohns disease
7. Frail individuals unable to tolerate a lower GI endoscopy
8. Previous surgery for colitis

Section 2 Demographic Data

Supplementary Table 1: Patient data – clinical information

	**Controls**	**LSPC**	**ROUC**	**PSC**	**DR**	**DT**	**ACT**§	**INACT**§
N	10	10	8	7	4	3	10	10
Age in years	60(41-75)	59.5(48-73)	31(22-52)	56(38-71)	60(20-74)	52(20-74)	36(23-71)	36(23-71)
Gender (Male:Female)	3:7	8:2	3:5	5:2	2:2	1:2	3:7	3:7
Duration of disease in years	NA	29 (21-42)	1.5 (0-4)	17 (5-35)	21 (9-41)	10 (9-41)	7 (1-45)	7 (1-45)
Baron Score	NA	0(0-1)	1(0-1)	0(0-2)	0(0-1)	NA	2(2-3)	0(0)
Histological activity index[24]	NA	0 (0-1)	0 (0-1)	0 (0)	0 (0)	NA	2 (1-3)	0 (0)

§ All data described as median and range (in parenthesis) unless mentioned

NA- not applicable; LSPC- Long standing pancolitis (>20 years disease duration); ROUC- Recent onset UC (<5 years disease duration); PSC- primary sclerosing cholangitis associated colitis; ACT- rectal biopsies from patients with actively inflamed distal colitis; INACT- biopsies proximal un-inflamed segment from patients in ACT group; DT- biopsies from the dysplastic segment;DR- biopsies from rectum in patients with dysplasia elsewhere in the colon.

Section 3 Cell Culture and IF extraction

Cell cultures

The breast adenocarcinoma cell line, MCF-7 was obtained from American Type Culture Collection (LGC Standards GmbH, Wesel, Germany). Cytoskeletal extracts from MCF-7 was used as an internal control during western blotting. Monolayer cultures of MCF-7 cells were prepared in T75 flasks using RPMI 1640 media (GIBCO, Invitrogen Paisley, UK) containing 10% foetal calf serum (Biosera, Sussex, U.K.). The cell culture medium was supplemented with Bio-Whittaker Pen-Strep (5000U/ml of each of Penicillin and Streptomycin, Lonza, Verviers, Belgium).

Cell fractionation and insoluble intermediate filament extraction from colonic biopsies and MCF-7 cell lines

IF extracts from cultured cells used as controls for reference and loading were prepared as described previously[29],

We have recently described and validated a technique for isolation and solubilisation of insoluble IFs from colorectal biopsies to enable parallel analyses by mass spectrometry (MS) and western immunolotting[29]. Briefly, colonic biopsies were processed sequentially in low and high detergent buffers (LDB and HDB) by a process of homogenization and centrifugation to yield the insoluble cytoskeleton fraction. This fraction was dissolved in 4M guanidine hydrochloride (GuHCl) (Sigma-Aldrich, Dorset UK) dissolved in triethyl ammonium bicarbonate buffer (TEAB) at a pH of 8.5 (Sigma-Aldrich, Dorset UK). Extraction of insoluble IFs from MCF-7 was undertaken as previously described[29].

Assay of protein concentration in GuHCl solubilised samples

The insoluble IF pellet obtained from the colonic biopsies were solubilized in 20µL of 4M GuHCl in TEAB. Protein concentration in solubilised cytoskeletal proteins was assayed in triplicate using Costar 96 well vinyl assay plates (Costar, Cambridge, MA) and Bio-Rad protein assay reagent (Bio-Rad Laboratories GmbH, Munchen, Germany) according to manufacturer's instructions. Absorbance was measured at 595 nm (reference at 450 nm) against standard BSA curves using a BioTek FLx800 multi-detection reader (BioTek, Instruments, Winooski, VT, USA).

Presence of protein in the dissolved samples was confirmed and appropriate protein loading in the SDS-PAGE gels was ensured by staining the gels with Coomassie blue.

Section 4 Proteomic Methods

A 8 plex iTRAQ analysis of proteins

Graphical Workflow summary of iTRAQ based protein profiling of IF fraction from colonic biopsies

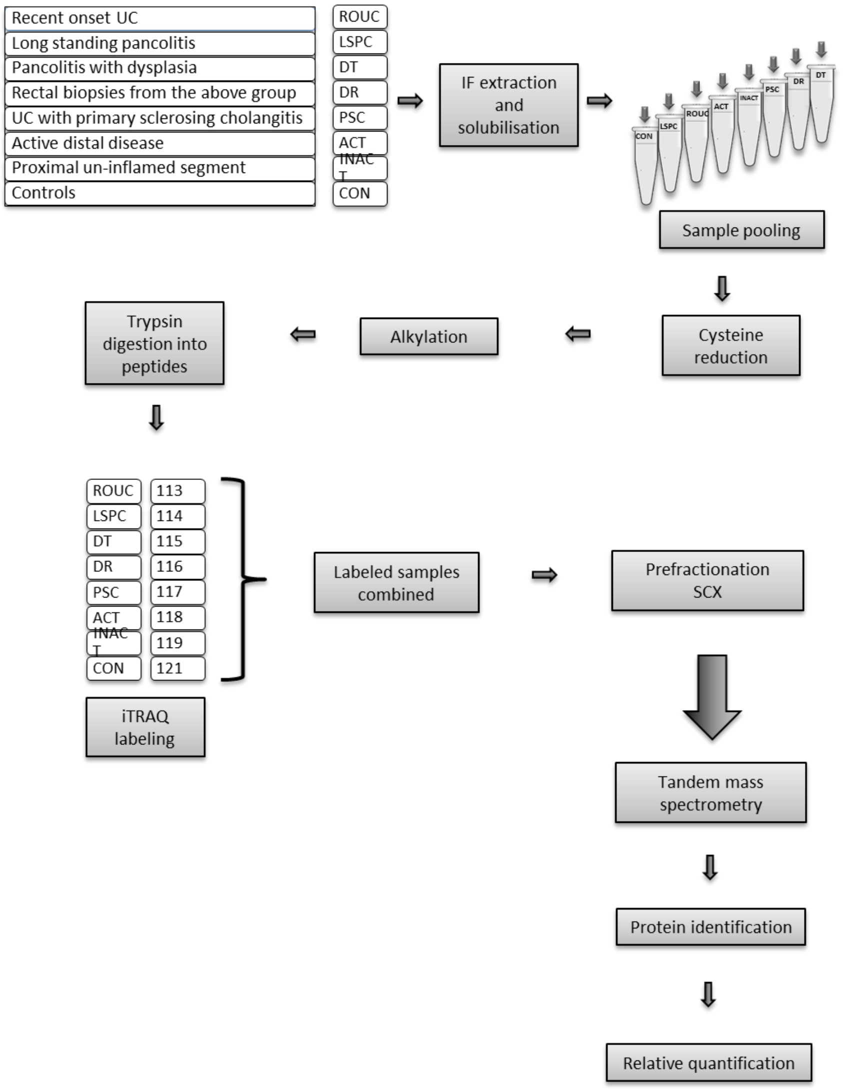

Figure 1. Experimental workflow.

Eight pooled samples from the experimental groups (CON- control, LSPC- long standing pancolitis, ROUC- recent onset UC, PSC- UC associated with primary sclerosing cholangitis, DR- rectal biopsy in patients with dysplasia, DT- biopsies obtained from the dysplastic tissue, ACT- active disease, INACT- proximal inactive segment in the ACT patients) were processed. An iTRAQ-compatible extraction and solubilisation protocol for insoluble intermediate filament proteins was developed and validated. Labelled peptides from pooled patients were analysed by SCX-LC-MS/MS (strong cation exchange-reverse phase HPLC tandem mass spectrometry) and data reconstituted in GeneBio Phenyx. All runs were searched against Uniprot database. Inter-group comparisons were made using in-house algorithms based on t-testing with multiple test correction

iTRAQ labeling

The dissolved cytoskeletal fraction obtained from individual colon biopsy samples were pooled into the respective eight groups being investigated as described in Supplemental figure 1. 100 µg of protein pooled in each group was diluted with TEAB to 2M GuHCl to ensure equal volumes for trypsin digestion. The cysteine residues in the samples were blocked by adding 50mM Tris (2-carboxyethyl) phosphine hydrochloride (TCEP-HCl) (Thermo Scientific, Rockford, IL, USA) followed by alkylation using 10mM Methyl methanethiosulfonate (MMTS) (Thermo Scientific, Rockford, IL, USA). Trypsin digestion was carried out at a 1:20 ratio for 16 hours at 37°C using sequencing grade trypsin (Promega, Madison, WI, USA) according to the manufacturers' instructions. Isopropanol (50 µl) was added separately to the 8 iTRAQ reagents (113-119, 121) (Applied Biosystems). The resulting digested peptides were iTRAQ labeled with 8-plex iTRAQ reagents (Figure 1) according to manufacturers' protocols (Applied Biosystems, CA, USA) as previously published[1].

The reagents were then added to the trypsin digested samples as follows:

113 : Controls (CON)

114: Active disease (ACT)

115: Inactive disease proximally (INACT)

116: Long standing Pancolitis (LSPC)

117: Recent onset UC (ROUC)

118: PSC and colitis (PSC)

119: Patients with dysplasia- biopsies obtained from the rectum (DR)

121: Biopsies from the dysplastic lesions (DT)

iTRAQ labeled samples were incubated at room temperature for 2 hours followed by pooling in a 1:1 ratio. The pooled mixture was dried in a vacuum centrifuge (Eppendorf Concentrator 5301) and was reconstituted in 90 µl of 20% acetonitrile (ACN), 0.1% formic acid and the pH of the solution was adjusted to 2. This reconstituted peptide mixture underwent sonication followed by centrifugation to remove insoluble material.

Strong Cation Exchange Fractionation of Peptides

Briefly, SCX was carried out using a PolySULFOETHYL™ A Column (PolyLC, Columbia, MD) 5µm particle size of 200mm length×4.6mm id, 200Å pore size, on a BioLC HPLC unit (Dionex, Surrey, UK). The 60-min gradient was generated between Buffer A (20% v/v acetonitrile,

0.1% v/v formic acid), and Buffer B (20% acetonitrile and 500mM KCl, 0.1% v/v formic acid), and consisted of 100% A for 5min, 5–30% B for 40 min, 30–100% B for 5min, 100% B for 5 min and finally 100% A for 5min. The chromatogram was monitored through a UV Detector (Dionex/LC Packings, Amsterdam, the Netherlands), at a wavelength of 214nm. Fractions were collected every minute and were later pooled together according to variations in peak intensity. SCX fractions were dried in a vacuum concentrator, and stored at −20°C prior to mass spectrometric analysis.

Tandem mass spectrometry (MS/MS) Analysis

An Ultimate 3000 capillary nano-LC (Dionex, LC Packings, The Netherlands) HPLC (high performance liquid chromatography) system was interfaced in tandem to reverse phase HPLC on line to a QSTAR® XL hybrid MS/MS System (ABSciex, Foster City, USA). Vacuum dried fractions obtained following offline separation techniques were re-suspended in loading buffer containing 3% acetonitrile (Sigma-Aldrich, Dorset UK) and 0.1% trifluoroacetic acid (Sigma-Aldrich, Dorset UK), and then injected and captured into a 0.3 x 5 mm trap column (3 µm C18 Dionex-LC Packings). An automated binary gradient set at a flow of 300 nL/min from 95% buffer A (3% acetonitrile, 0.1% formic acid), to 35% buffer B (97% acetonitrile, 0.1% formic acid) over 90 min, followed by a 5 min ramp to 95% buffer B (with isocratic washing for 10 min) was used to elute samples trapped in the columns onto a 0.075 x 150 mm analytical column (3 µm C18 Dionex-LC Packings). The mass spectrometer was set to perform data acquisition in the positive ion mode. Survey scans (350−1200 *m/z)* were acquired with up to two dynamically excluded precursors selected for MS/MS (*m/z* 65−1600). The collision energy range was raised to 20% higher than needed unlabeled peptides to overcome the stabilizing effect of the basic N-terminal derivative and to achieve equivalent fragmentation as recommend by Applied Biosystems.

iTRAQ data analysis

The mass-spectrometric data was collected and analysed as previously described **[3,** (Majumdar et al., 2012)**.** Briefly, MS/MS data generated from the QSTAR® XL was first converted to generic MGF peaklists using the mascot.dll embedded script (version 1.6 release no. 25) in Analyst QS v. 1.1 (Applied Biosystems, Sciex; Matrix Science). Further processing of the data was undertaken using an in-house Phenyx algorithm cluster (binary version 2.6; Geneva Bioinformatics SA) at the ChELSI Institute, University of Sheffield, against the *Homo sapiens* UniProt protein knowledgebase (SwissProt and Trembl (41070 and

71449 entries respectively, downloaded 5th November 2010,) to derive peptide sequence and hence protein identification. These data were then searched within the reversed *Homo sapiens* database to estimate the false-positive rate (6). Peptides identifications at 1% false discovery rate were accepted. The iTRAQ reporter ion intensities were exported. Protein quantifications were obtained by computing the geometric means of the reporters' intensities. Median correction was subsequently applied to every reporter in order to compensate for systematic errors, *e.g.* if a sample happened to have been loaded at a largely different total concentration. The reporters' intensities, in each individual MS/MS scan, were also median corrected using the same factors, with the rationale that if the total concentration of a sample A was half that of another sample B, the intensities of sample A's reporter have to be doubled to allow for a fair comparison. *t*-tests applied to determine alterations in protein level between samples use these corrected intensities since these were was carried out for every protein and because of the multiple times each test was performed, the threshold (α=5%) used for significance was corrected for data mining. Here, we used the standard Bonferroni correction (α/P, where P is the number of proteins) to minimise false positive results. This workflow was developed in house (3)

B Label free MS analysis

Complex tryptic peptide mixtures were separated using nanoscale chromatography performed using a nanoACQUITY UPLC (Waters Corporation, USA). One dimensional reversed phase (RP) nanoACQUITY experiments with trapping were performed. Mobile phases A and B were water containing 0.1% (v/v) formic acid and acetonitrile containing 0.1% (v/v) formic acid respectively. Following desalting of the peptides on a Symmetry C18 5 µm, 2 cm x 180 µm trap column, (Waters Corporation, USA) a reversed phase gradient was employed to separate peptides using 5 to 40% acetonitrile over 90 minutes on a 25 cm x 75 µm analytical RP column (Waters Corporation, USA) at a flow rate of 300 nL/min and a constant temperature of 35 ^{o}C.

Analysis of the peptides extracted from the gel bands was performed using a SYNAPT G2-Si HDMS mass spectrometer (Waters, Manchester UK) operated in a data-independent manner coupled with ion mobility ($HDMS^E$) (Rodriguez-Suarez, as per ASMS 2013). The mass spectrometer was operated in positive ESI resolution mode with resolution of >25,000 FWHM. In all experiments the mass spectrometer was programmed to step between low energy (4 eV) and elevated (14-40 eV) collision energies on the Triwave collision cell, using a scan time of 0.9 s per function over 50-2000 m/z. All samples were analyzed in duplicate.

Analysis of label free $HDMS^E$ data

Data processing and database searching was performed using and a UniProt human database using algorithm of Progenesis QI software as described by (Li et al., 2009, Proteomics 6:1696-1719 Database searching and accounting of multiplex spectra.)Validation and comparative analyses were carried out using Scaffold(version Scaffold_4.3.2, Proteome Software Inc., Portland, OR) was used to validate MS/MS based peptide and protein identifications. Peptide identifications were accepted if they could be established at greater than 95.0% probability by the Peptide Prophet algorithm (Keller, A et al Anal. Chem. 2002;74(20):5383-92). Protein identifications were accepted if they could be established at greater than 90.0% probability and contained at least 2 identified peptides. Protein probabilities were assigned by the Protein Prophet algorithm (Nesvizhskii, Al et al Anal. Chem. 2003;75(17):4646-58). Proteins that contained similar peptides and could not be differentiated based on MS/MS analysis alone were grouped to satisfy the principles of parsimony. Proteins were annotated with GO terms from gene_association.goa_human (downloaded Jul 22, 2013). (Ashburner, M et al Nat. Genet. 2000;25(1):25-9).

Section 5 SDS-PAGE and Western Transfer

Quantitative analysis of immunoblot was performed using densitometry. The Chemigenius Bio-Imaging System and Gene Tools software (Syngene, Cambridge, UK), were used. . Individual bands of interest were selected manually and their densities were calculated. The software was used to automatically correct any background intensity. Densitometry results to calculate the proportion of phosphorylated K8 were normalised to the MCF-7 internal standard and calculated as follows: Phosphorylated K8 band intensity/MCF-7 band intensity was calculated (x). Total K8 band intensity/MCF-7 band intensity was calculated (y). x/y would equate to the phosphorylated K8 ratio. Finally the results were transferred to Microsoft Excel (Microsoft Corporation, Redmond, USA).

Coomassie staining of the gels was performed separately using Instant Blue (Expedeon, Harston, UK) in order to visualise the protein bands.

Section 6 Proteomic Data

A graphical summary of the workflow, relating the pooling and unpooled analysis and tiers of orthogonal validation.

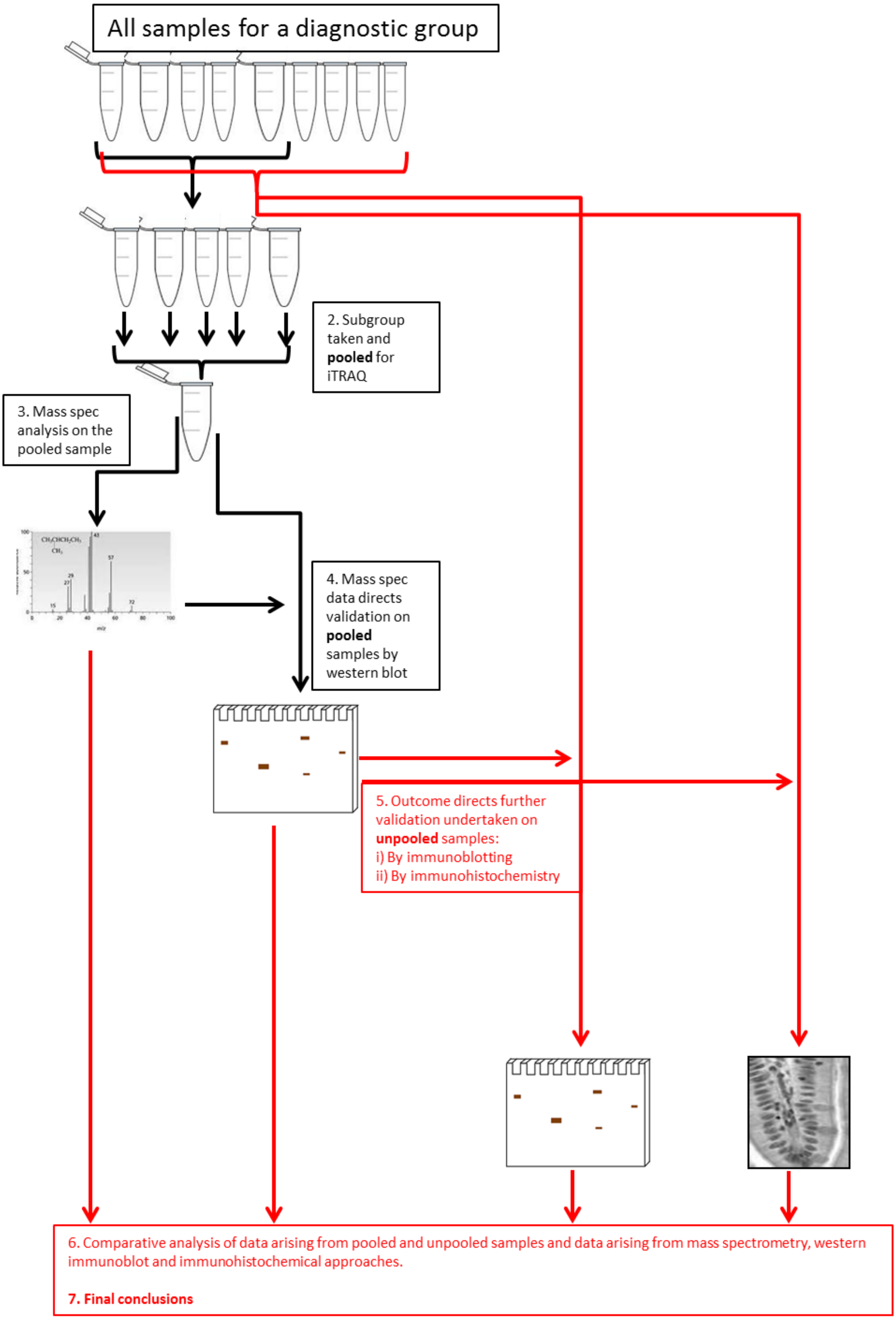

. Patient demographics pooled for the iTRAQ study;

**POOLED (iTRAQ)**	**Number of patients**	**Age**	**Sex**	**Disease extent**
**ACT**	10	Median:36<br>Range:23-71	F:8; M:2	Proctitis:5; Proctosigmoiditis:2; Lt sided:3
**INACT**	10	Median:36<br>Range:23-71	F:8; M:2	Proctitis:5; Proctosigmoiditis:2; Lt sided:3
**LSPC**	10	Median: 59.5<br>Range:48-73	F:2; M:8	Pancolitis:10
**ROUC**	8	Median:31<br>Range:22-52	F:5; M:3	Proctitis:1;<br>Proctosigmoiditis:1;<br>Lt sided colitis:2;<br>Pancolitis:4
**PSC colitis**	7	Median:56<br>Range:38-71	F:2; M:5	Pancolitis:7
**DR**	4	Median:68<br>Range:52-74	F:2; M:2	Pnacolitis:4
**DT**	3	Median:60<br>Range:20-74	F:2; M:1	Pancolitis:3
**CON**	10	Median:60<br>Range:41-75	F:6;M:4	N/A

Supplemental Table 2. Proteins identified and relatively quantified in the iTRAQ proteomic data set

Accession	# peptides	#valid peptide seq	% Coverage	Score	Mass (kDa)	Description
Q96KK5	78	3	17	25.1	13775	Histone H2A type 1-H
O60814	68	8	55	60.2	13759	Histone H2B type 1-K
B4E380	62	8	41	62.0	12918	Histone H3
Q53SW3	40	1	2	7.5	52094	Putative uncharacterized protein DPYSL5
P62805	38	6	47	48.1	11236	Histone H4 [CHAIN 0]
P05787	35	11	20	95.3	53573	Keratin, type II cytoskeletal 8
P08727	31	13	25	122.0	44092	Keratin, type I cytoskeletal 19
P30989	24	1	2	5.4	46259	Neurotensin receptor type 1
Q147W7	23	1	3	11.0	37522	KRT1B protein
P05783	17	4	7	38.1	47927	Keratin, type I cytoskeletal 18
D3DP13	15	2	4	16.0	39737	Fibrinogen beta chain
P12111	15	19	6	138.3	321350	Collagen alpha-3(VI) chain
P08123	13	4	3	29.3	91755	Collagen alpha-2(I) chain
B4E241	6	1	7	9.4	14203	Splicing factor, arginine/serine-rich 3
O75084	5	1	1	6.6	60730	Frizzled-7
P11678	5	1	2	7.4	53422	Eosinophil peroxidase heavy chain
Q702N8	5	1	1	7.2	122134	Xin actin-binding repeat-containing protein 1
Q9NRC6	5	2	0	12.7	416835	Spectrin beta chain, brain 4
B3KRK8	5	4	6	37.2	46976	cDNA FLJ34494 fis, highly similar to VIMENTIN
B4DJC3	4	1	5	9.3	21548	Histone H2A
P07355	4	2	7	16.2	38473	Annexin A2
P59665	4	2	35	16.0	3377	Neutrophil defensin 2

Q9HAM5	4	1	3	8.8	21598	cDNA FLJ11359 fis, moderately similar to HYPOXIA-INDUCIBLE FACTOR 1 ALPHA;
B4DUR6	3	1	1	7.0	76094	cDNA FLJ58493, highly similar to Centaurin-gamma 2
P12644	3	1	2	6.0	46555	Bone morphogenetic protein 4
Q68CJ9	3	1	2	7.0	44153	Processed cyclic AMP-responsive element-binding protein 3-like protein 3
Q9H9P2	3	1	3	6.4	25979	Chondrolectin
D3DWL0	3	2	1	12.2	234119	Plectin 1, intermediate filament binding protein 500kDa
Q5T1W7	2	1	3	7.5	21473	Beta-transducin repeat containing
Q6ZNC2	2	1	3	6.1	30533	cDNA FLJ16223 fis, clone CTONG3003598
B3KR37	2	1	1	5.7	55951	cDNA FLJ33617 fis, clone BRAMY2019055, highly similar to Epidermal growth factor receptor substrate 15
B4DVQ0	1	5	13	42.0	37349	cDNA FLJ58286, highly similar to Actin, cytoplasmic 2
C9JLJ5	1	1	5	6.1	16083	Putative uncharacterized protein TRIP12
D3DX73	1	1	1	6.2	101590	HCG1994636, isoform CRA_b
P04053	1	1	2	5.7	58309	DNA nucleotidylexotransferase
P52961	1	1	3	6.8	30538	GPI-linked NAD(P)(+)--arginine ADP-ribosyltransferase 1
Q03167	1	1	1	7.3	91316	Transforming growth factor beta receptor type 3
Q13032	1	1	2	6.7	80137	Transporter
Q14118	1	1	4	5.9	26557	Beta-dystroglycan
Q15161	1	1	5	6.1	12747	DNA-directed RNA polymerase
Q59GU6	1	1	2	6.4	50099	Sorting nexin 1 isoform a variant
Q5CZ99	1	1	1	6.8	85623	Putative uncharacterized protein

						DKFZp686I1370
Q5T911	1	1	4	5.3	27077	Mediator complex subunit 4
Q5TEC6	1	5	29	38.1	15430	Histone H3
Q5VZM2	1	1	2	5.7	40172	Ras-related GTP-binding protein B
Q6ZSP3	1	1	1	8.5	133571	cDNA FLJ45330 fis, highly similar to Potential phospholipid-transporting ATPase IB
Q8TE58	1	1	1	5.9	80515	A disintegrin and metalloproteinase with thrombospondin motifs 15
Q8TES4	1	2	1	12.8	153599	FLJ00119 protein
Q96Q15	1	1	0	6.8	269425	Serine/threonine-protein kinase SMG1
Q9HDB4	1	1	6	6.0	13200	Putative uncharacterized protein CATX-1

Section 7. Summary of densitometric analyses of western blot data

Alterations in ratio of intermediate filament proteins VIM and phosphorylated K8 relative to total K8 in colonic mucosa in acute inflammation**.**

3A. In the acutely inflamed mucosa there is a reduction in both VIM as well as total K8 levels; the VIM:K8 ratio is thus markedly low in acute inflammation. A relative increase (in comparison to controls) in total K8 is seen in the un-inflamed mucosa. This is reflected in an increased VIM:K8 ratio in the healthy controls relative to un-inflamed proximal mucosa;

3A-C. In general phosphorylated K8 is reduced or absent (K8pS23) in presence of acute inflammation.

A. VIM:K8 ratio

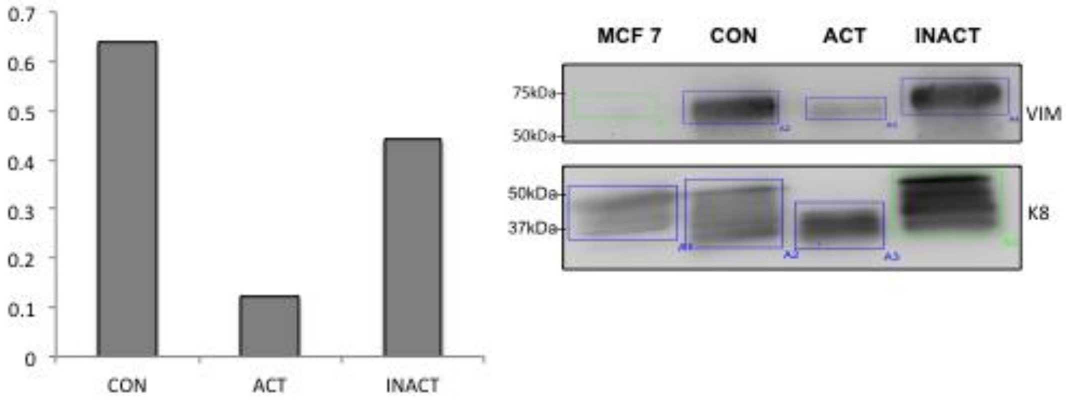

B. K8pSer^{23}:Total K8 ratio

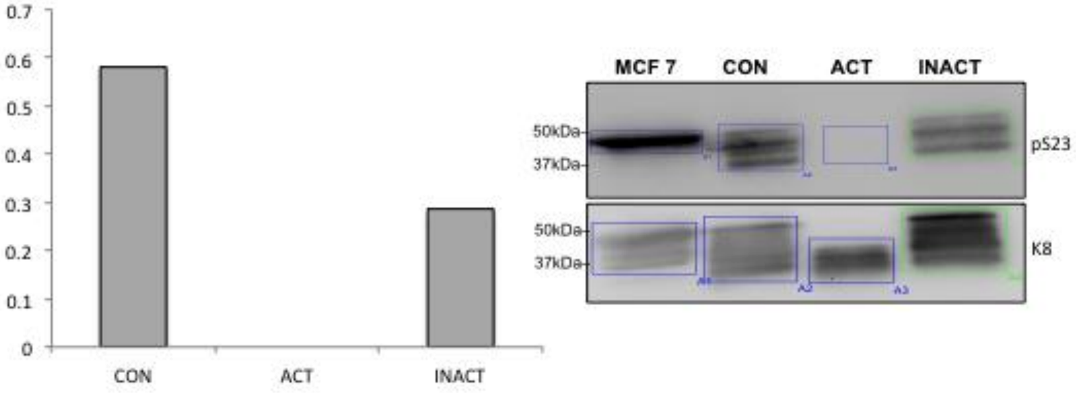

C. K8pSer^{73}:Total K8 ratio

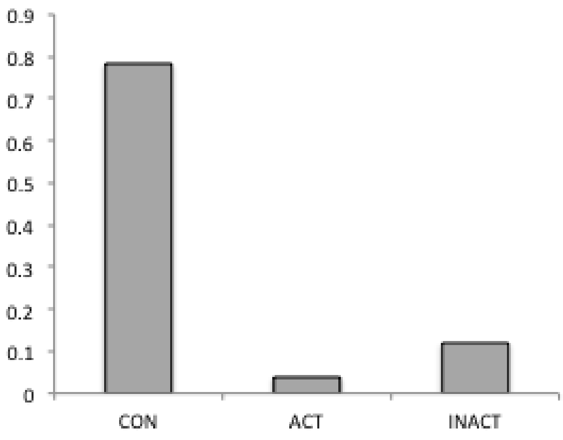

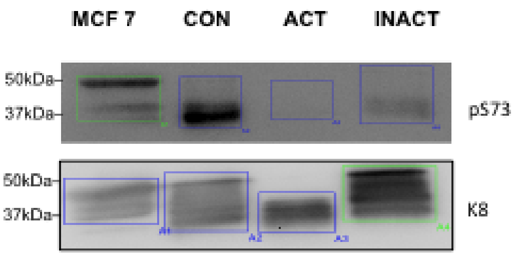

D. K8pS^{431}:Total K8 ratio

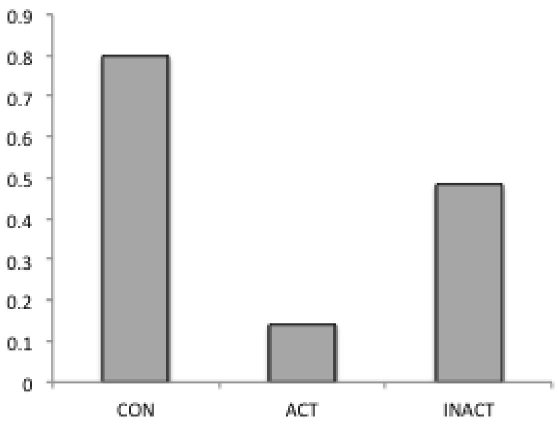

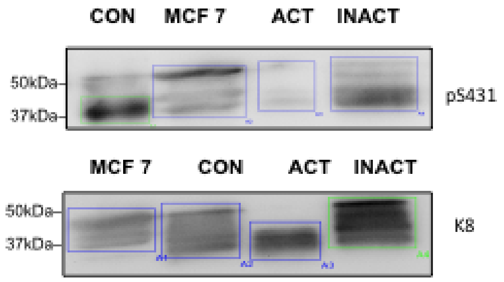

E: K8 Phosphorylations by Patient Group

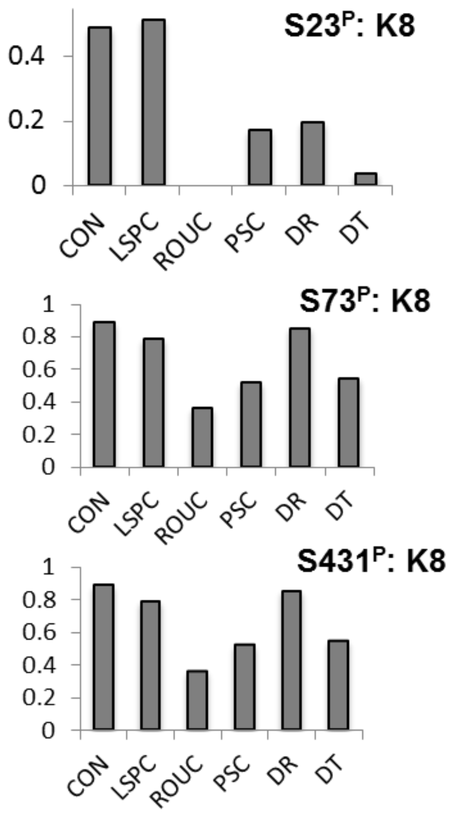

Section 8. Analysis of unpooled samples

Patient demographics for the validation experiments with western blotting (WB) for K8, K18 & K19 on unpooled samples used in the pooling

POOLED (WB)	Number of patients	Age	Sex	Disease extent
**ACT**	10	Median:36<br>Range:23-71	F:8; M:2	Proctitis:5<br>Proctosigmoiditis:2<br>Lt sided:3
**INACT**	10	Median:36<br>Range:23-71	F:8; M:2	Proctitis:5<br>Proctosigmoiditis:2<br>Lt sided:3
**LSPC**	10	Median: 59.5<br>Range:48-73	F:2; M:8	Pancolitis:10
**ROUC**	8	Median:31<br>Range:22-52	F:5; M:3	Proctitis:1<br>Proctosigmoiditis:1<br>Lt sided colitis:2<br>Pancolitis:4

. Patient demographics for the cross-validation experiments with western blotting (WB) for K8, K18 & K19 on a new cohort

Unpooled (WB)	Number of patients	Age	Sex	Disease extent

**ACT**	7	Median:36<br><br>Range:23-71	F:3; M:4	Proctosigmoiditis:1<br>Lt sided colitis:6
**INACT**	7	Median:36<br><br>Range:23-71	F:3; M:4	Proctosigmoiditis:1<br>Lt sided colitis:6
**CON**	4	Median:54<br><br>Range:43-57	F:3;M:1	N/A

Table 4. Patient demographics for the validation and cross-validation experiment with immunohistochemistry (IHC) for K8 antibody on a mix of samples used in pooling as well as new sample cohorts;

Mixed (IHC, K8)	Number of patients	Age	Sex	Disease extent
**ACT**	11	Median: 45<br><br>Range: 23-71	F: 7; M: 4	Proctitis:3<br><br>Proctosigmoiditis:1<br><br>Lt sided colitis:4<br><br>Pancolitis:3
**INACT**	11	Median: 45<br><br>Range: 23-71	F:7; M:4	Proctitis:3<br><br>Proctosigmoiditis:1<br><br>Lt sided colitis:4<br><br>Pancolitis:3

**LSPC**	4	Median: 59.5<br>Range: 56-73	M:4	Pancolitis:4
**ROUC**	7	Median: 31<br>Range: 22-52	F:4;M:3	Proctosigmoiditis:1<br>Lt sided colitis:2<br>Pancolitis:4
**PSC colitis**	5	Median: 58<br>Range: 44-71	F:1; M:4	Pancolitis:5
**DR**	1	20	M:1	Pancolitis:1
**DT**	1	52	F:1	Pancolitis:1
**CON**	8	Median:59<br>Range: 53-77	F:5; M:3	N/A

8.1 Immunoblotting of unpooled sample for active versus inactive regions

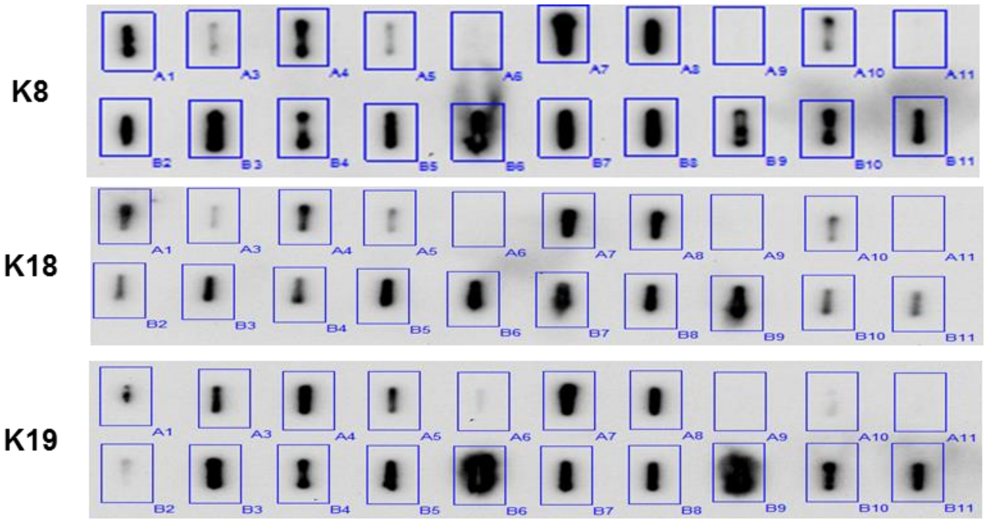

8.2 Immunoblotting for Keratins 8, 18 and 19 comparing individual colonic biopsies from patients with active colitis (ACT) with the reciprocal un-inflamed proximal inactive mucosa (INACT). MCF-7 cell line as well as sigmoid biopsy from a single normal individual was used as controls. A1: MCF-7; B2: Sigmoid biopsy from a normal control; ACT: A3-A11; INACT: B3-B11.

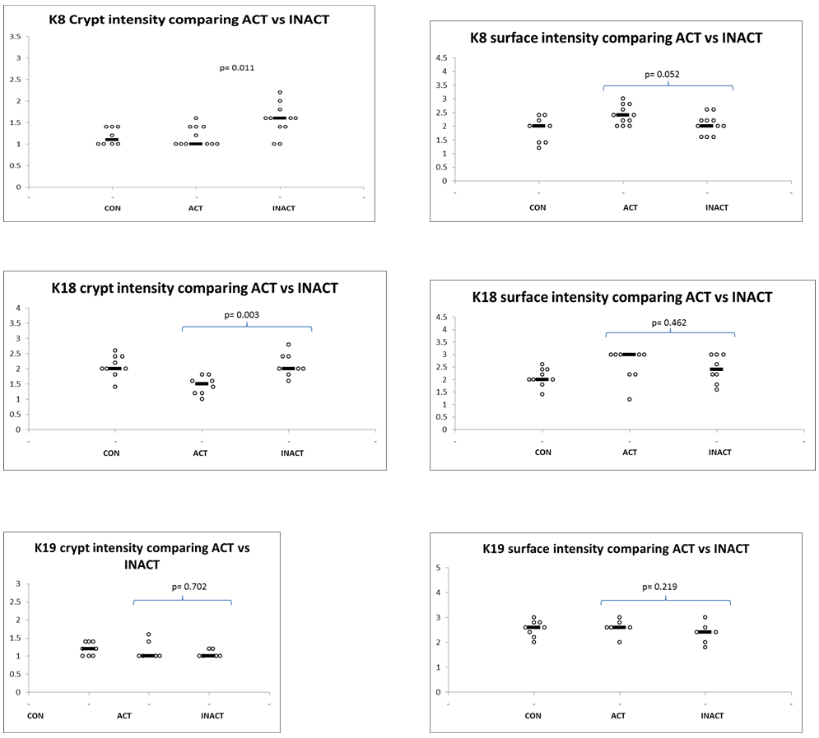

Immunohistochemical scores of biopsies for intensity of surface and crypt staining of K8, K18 and K19.
